# A haplotype in CFH family genes confers high risk of rare glomerular nephropathies

**DOI:** 10.1038/s41598-017-05173-8

**Published:** 2017-07-20

**Authors:** Yin Ding, Weiwei Zhao, Tao Zhang, Hao Qiang, Jianping Lu, Xin Su, Shuzhen Wen, Feng Xu, Mingchao Zhang, Haitao Zhang, Caihong Zeng, Zhihong Liu, Huimei Chen

**Affiliations:** 1 Devision of Nephrology, Jinling Hospital, Southern Medical University, Nanjing, 210016 China; 2 National Clinical Research Center of Kidney Diseases, Jinling Hospital, Nanjing University School of Medicine, Nanjing, 210016 China; 3 Center of Drug Discovery, State Key Laboratory of Natural Medicines, China Pharmaceutical University, Nanjing, 210009 China

## Abstract

Despite distinct renal lesions, a series of rare glomerular nephropathies are reportedly mediated by complement overactivation. Genetic variations in complement genes contribute to disease risk, but the relationship of genotype to phenotype has not been straightforward. Here, we screened 11 complement genes from 91 patients with atypical hemolytic uremic syndrome (aHUS), C3 glomerulopathy (C3G) and membranoproliferative glomerulonephritis type I (MPGN I), and identified the concomitant presence of three missense variations located within the human complement Factor H (CFH) gene cluster. The three variations, rs55807605, rs61737525 and rs57960694, have strong linkage disequilibrium; subsequent haplotype analysis indicated that ATA increased the susceptibility of these renal diseases. In silico analysis, the CFHR3 rs61737525-T risk allele altered the physical and structural properties and generated a reduction in binding affinity of the CFHR3/C3b complex. Surface plasmon resonance (SPR) binding analysis further demonstrated the substitution induced a decrease of two orders of magnitude in C3b-binding properties, with a declined cofactor activity in fluid phase. These data suggest that the haplotype carrying the causative allele behaves as a partial C3 convertase deficiency, predisposing individuals to diverse pathologic lesions underlying complement overactivation. Such genotype-phenotype discrepancies allow better understanding about these nephropathies mediated by genetic complement disorders.

## Introduction

The complement cascade is part of the innate immune system and provides an important line of defense against invasive pathogens. Its key step is the cleavage of C3 to C3a and C3b affected by C3 convertase activity, and the latter may originate from the classic, lectin or alternative pathway (AP)^1, 2^. The complement system has long been recognized as having a role in immune complex-mediated glomerulonephritis, and the pattern of complement activation is via the classic complement pathway. Recently, the AP has also been suggested to cause kidney injury in a wide spectrum of diseases. Excessive complement activation is particularly important in the pathogenesis of atypical hemolytic uremic syndrome (aHUS), membranoproliferative glomerulonephritis type I (MPGN I), and C3 glomerulopathy (C3G); the latter includes dense deposit disease (DDD) and C3 glomerulonephritis (C3GN)^3–5^.

Increasing association of genetic variations in complement and complement control proteins was observed in patients with these rare renal diseases. Complement factor H (CFH) is an important molecule controlling the complement system, regulating the complement activation in several ways. Mutations in the CFH gene are associated with a number of infectious and inflammatory conditions, which include an increased tendency for MPGN I and aHUS, as well as C3G^6–11^. Similar alterations were found to be involved in the distinct renal pathology of these rare kidney diseases. The homozygous Pro621Thr in CFH was first detected as a causative mutation to be associated with undetectable circulating CFH levels in a patient who first developed C3G and later showed a shift to aHUS^12^. The Tyr899Stop in CFH was found at homozygosity in a patient with CFH deficiency and aHUS, who has been documented to develop C3G after kidney transplantation^13^. Raychaudhuri *et al*. identified a rare, high-risk CFH mutation (Arg1210Cys) in age-related macular degeneration (AMD), which was previously detected in aHUS and C3G^14–16^.

Genetic complement deficiencies are responsible for around 20~60% of these rare glomerular nephropathies^3, 4^. Due to lack of specialized diagnostic laboratories, this number is likely an underestimate. Sequencing of the genes coding for complement components has long been used to investigate the mechanism behind these manifestations. Understanding the effect of genetic background on mutant phenotypes also has specific medical relevance. Here, we reported three rare single nucleotide polymorphisms (SNPs) located within the CFH gene cluster by high-throughput sequencing for 11 complement genes. They were identified to be causative variations, which have been associated with renal patients with aHUS, DDD, C3GN or MPGN I. Furthermore, the pathologic significance was systematically evaluated in the present study.

## Results

**Figure 1:**
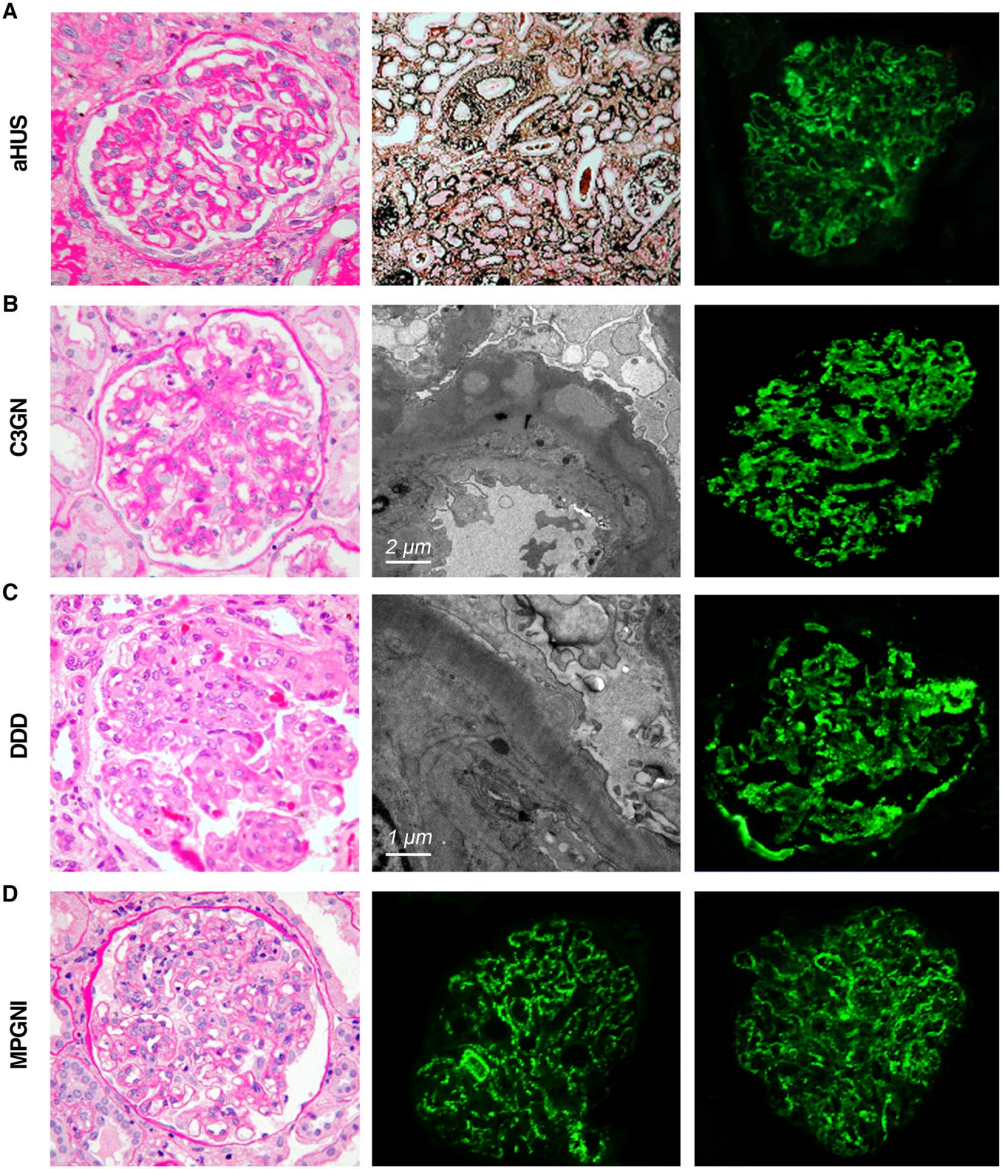
Representative light, electron microscopy and immunofluorescence in cases of aHUS, C3GN, DDD and MPGNI, respectively. The aHUS patient demonstrated a mild mesangial proliferation and arterial thrombotic microangiopathy (TMA) with obliteration of capillary lumina under the light microscope. The C3GN patient showed a moderate to severe mesangial proliferation by light microscope, as well as subepithelial and intramembranous electron-dense deposits by electron microscopy. The DDD patient displayed diffuse endothelial and mesangial cell proliferation with hyaline thrombus formation in capillary loops in the light microscope and ribbon-like high electron-dense intramembranous deposits in the electron microscopy. The MPGN I patient exhibited a moderate mesangial proliferation and diffuse endothelial cell proliferation under the light microscope. A marked C3 staining was detected to locate along the capillary wall or mesangium by immunofluorescence analysis among all of them, with dominant IgG deposition along the capillary wall in the patient with MPGNI.

After gene screening, we focused on three rare SNPs, rs55807605 (CFH c.2509 G > A), rs61737525 (CFHR3 c.424 C > T) and rs57960694 (CFHR5 c.434 G > A), which were simultaneously detected in four cases (case 1, case 2, case 3 and case 4). All of them were sporadic cases and diagnosed with aHUS, G3GN, DDD and MPGN I, respectively (Supplementary Table S5). The patients with aHUS and C3GN presented a low level of C3, while the other two patients were in the normal range during the disease period. Representative renal lesions are shown in Fig. 1. These lesions had a marked C3 staining along the capillary wall or mesangium detected by immunofluorescence analysis. The patient with MPGNI had a dominant IgG deposition along the capillary wall.

### Genetic findings

**Figure 2:**
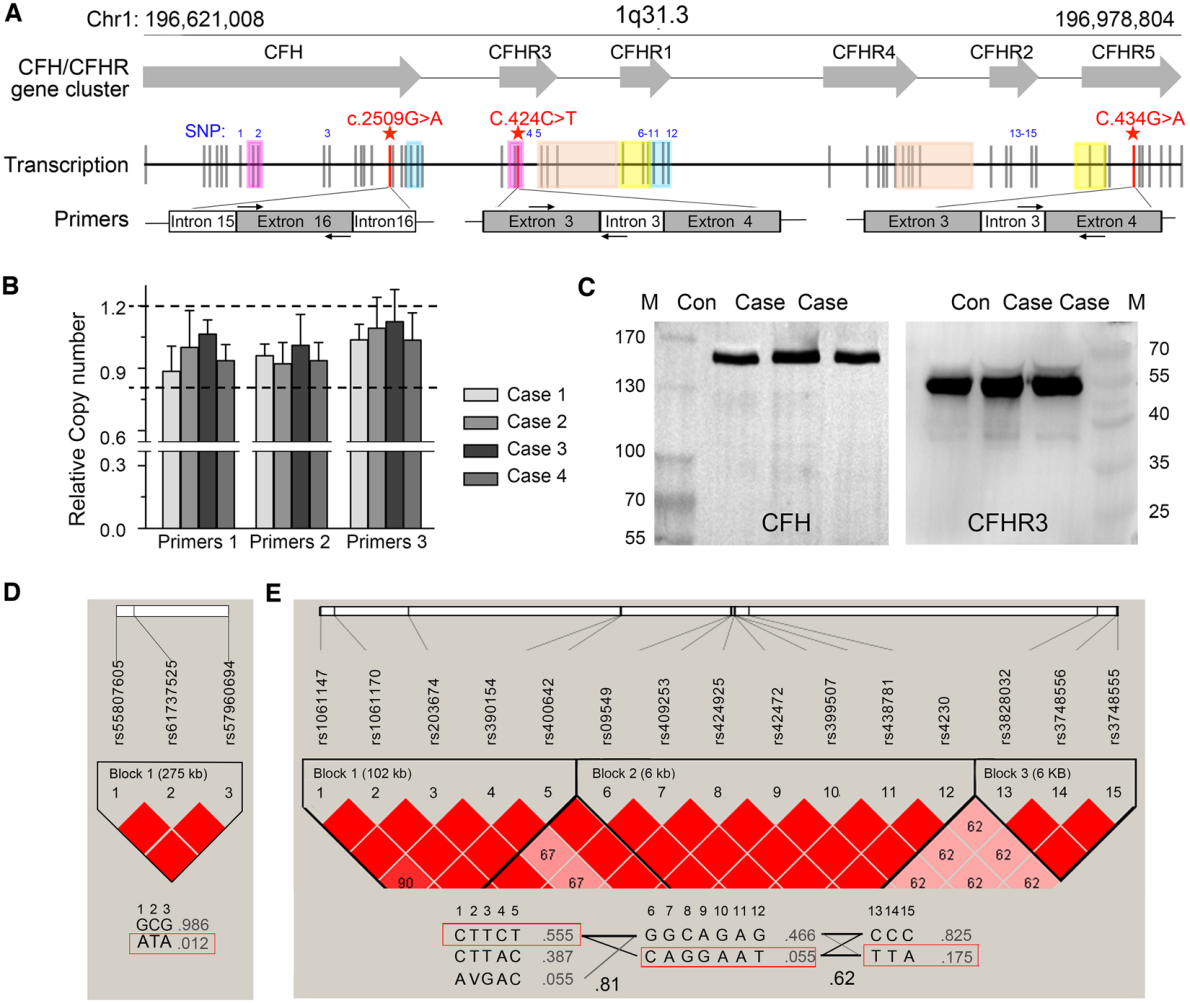
Genetic analysis of three rare SNPs and other 15 common SNPs. (**A**) Locations of rare rs55807605 (CFH c.2509 G > A), rs61737525 (CFHR3 c.424 C > T) and rs57960694 (CFHR5 c.434 G > A) are presented with red asterisk, corresponding to three pairs of primers specific of RS gene loci, while 15 other common SNPs are in blue number notation. Genomic duplications including the different exons of CFH, CFHR3 and CFHR5 are color-coded. Exons are indicated as vertical lines. (**B**) Relative copy number of three fragments of CFH, CFHR3 and CFHR5 linked with these SNPs in patients and controls measured by real-time quantitative PCR and T-tests were used to quantify significant differences between patients and controls (P < 0.05). (**C**) Western blotting bands of CFH and CFHR3 protein among cases and healthy controls. Linkage disequilibrium plots show that (**D**) three rare SNPs and (**E**) 15 common SNPs are all in linkage disequilibrium in the CFH gene cluster (n = 208; Chinese Han population). Haplotype frequencies and crossover frequencies between blocks are shown in the schematic, respectively.

CFH, CFHR3 and CFHR5 are members of the human complement Factor H gene cluster, located at 1q31.3 (GRCh37, Fig. 2A), which is enriched with large genomic repeat regions. To exclude potential genetic fusion linked with these SNPs, we evaluated the relative copy numbers of three fragments at 1q31.3 to the GAPDH gene in the range of 159 bp to 187 bp (Fig. 2B). Compared with controls, i.e., cases showing similar copy numbers in this region, indicating the absence of duplication/deletion across the involved fragments. Western blotting analysis further confirmed the same bands of CFH and CFHR3 proteins among cases and healthy controls (Fig. 2C). Only one band was found around the 150-kDa or 55-kDa component on the SDS-PAGE for CFH and CFHR3, respectively. No additional bands were observed.

Linkage disequilibrium (LD) structure was analyzed based on the Chinese Han population (1000 Genomes Project Phase 3). Rs55807605, rs61737525 and rs57960694 showed strong LD (all r^2^ > 0.8, D’ = 1 as per Fig. 2D) which constructed two haplotypes (“GCG” and “ATA”), and the four patients carrying the three combined SNPs shared a rare ATA haplotype (1.2%). When 15 other polymorphisms were used to analyze the LD across the CFH gene cluster, it confirmed strong LD with 3 blocks (Fig. 2E), and the four patients carried the same haplotype in each block, with CTTCT, CAGGAAT and TTA, respectively.

### Risk association of rs55807605, rs61737525 and rs57960694

Since the three SNPs were first identified among the cohort of 91 rare nephropathies, we further randomly enrolled a matched control cohort, containing 300 enrolled healthy subjects. As shown in Table 1, CFHR3 c.424 C > T (rs61737525) significantly increased the risk for rare nephropathies compared with healthy controls, with an odds ratio (OR) of 4.21 (95% CI = 1.12–15.84 P = 0.036). The dominant model analysis further showed an OR of 6.85 (95% CI = 1.23–38.03, P = 0.028). But, rs55807605 and rs57960694 presented no direct association with these glomerular diseases.

**Table 1:** Comparison of genotype and allele frequencies of CFH, CFHR3 and CFHR5 single nucleotide polymorphisms (SNPs) in patients with rare glomerular nephropathies and control subjects.

SNP ID		Cases (n = 91)	Controls (n = 300)	P-value*	OR (95% CI)
CFH					
rs55807605					
Allele (%)	A	4 (2.2)	10 (1.7)	0.749	1.33 (0.41–4.28)
	G	178 (97.8)	590 (98.3)		
Genotype (%)	AA	0 (0.0)	0 (0.0)	0.747^a^	1.33 (0.41–4.36)
	AG	4 (4.4)	10 (3.3)		
	GG	87 (95.6)	290 (96.7)		
CFHR3					
rs61737525					
Allele (%)	T	5 (2.7)	4 (0.7)	0.036	4.21 (1.12–15.84)
	C	177 (97.3)	596 (99.3)		
Genotype (%)	TT	1 (1.1)	2 (0.7)	0.028^a^	6.85 (1.23–38.03)
	TC	3 (3.3)	0 (0.0)	0.549^b^	1.66 (0.15–18.47)
	CC	87 (95.6)	298 (99.3)		
CFHR5					
rs57960694					
Allele (%)	A	4 (2.2)	4 (0.7)	0.090	3.35 (0.83–13.52)
	G	178 (97.8)	596 (99.3)		
Genotype (%)	AA	0 (0.0)	0 (0.0)	0.089^a^	3.40 (0.83–13.89)
	AG	4 (4.4)	4 (2.3)		
	GG	87 (95.6)	296 (97.7)		

n = number of subjects; OR = odds ratio; CI = confidence interval. Data shown are the number of subjects (% of the total group). *Fisher’s exact text.

^a^P value for dominant model; ^b^P value for recessive mode.

Haplotype-association analysis further revealed that the ATA haplotype of the three minor alleles (A at rs55807605, T at rs61737525, and A at rs57960694) was significantly associated with increased risk (P = 0.029; OR, 6.72; 95% CI, 1.22–36.99) (Table 2). This haplotype was described completely by the allele T at rs61737525, but its haplotypic P value did increase with statistical significance compared with the single-allele analysis of rs61737525. CFH, CFHR3 and CFHR5 proteins all regulate AP activation, but no significant synergistic interaction of these three SPNs was observed under nonparametric MDR (Supplemental Figure S1). These data suggested that c.424 C > T in CFHR3 (rs61737525) was the leading pathogenic variant in this risk gene locus.

**Table 2:** Haplotype analysis for CFH, CFHR3 and CFHR5 single nucleotide polymorphisms (SNPs).

SNPs alleles			Haplotype frequency		Association test between rare kidney diseases and controls	
rs55807605	rs61737525	rs57960694	Cases (n = 91)	Controls (n = 300)	P-value*	OR (95% CI)
G	C	G	0.973	0.980	0.563	0.72 (0.25–2.08)
A	C	G	0.000	0.010	0.345	1.010 (1.002–1.018)
A	T	A	0.022	0.003	0.029	6.72 (1.22–36.99)
G	T	G	0.005	0.003	0.549	1.65 (0.15–18.32)
A	C	A	0.000	0.003	1.000	1.003 (0.999–1.008)

n = number of subjects; OR = odds ratio; CI = confidence interval.

Individual P values and ORs between cases with rare kidney diseases and controls are provided for the haplotypes

compared with all the other haplotypes. *Fisher’s exact text.

### Pathogenicity prediction for CFHR3 c.424 C > T (rs61737525)

**Figure 3:**
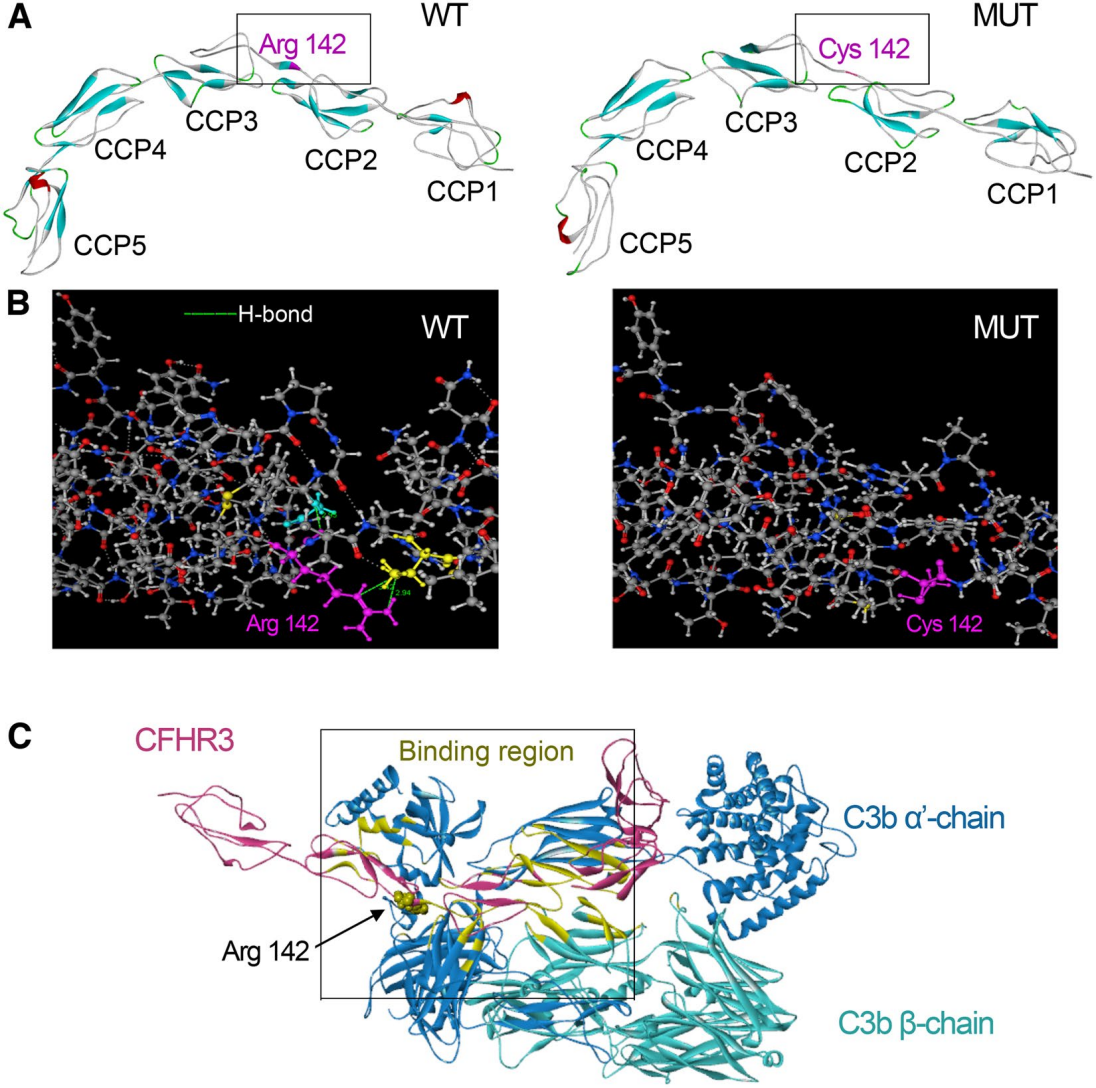
Three-dimensional models of CFHR3_WT_, CFHR3_Arg142Cys_ and CFHR3/C3b complex. (**A**) Models of WT and MUT CFHR3 obtained by homology-modeled and shown with 5 complement control protein modules (CCPs) by DS 3.0. The β-strands are shown in turquoise, а-helices in red, and the loops connecting in WhiteSmoke™. The location of the Arg142 and Cys142 is highlighted in orchid. (**B**) Generated models show the discrimination of local intramolecular hydrogen bonding interactions between Arg142 and Cys142 by MOE. Hydrogen bonds are shown with a fluorescent green dotted line representation. (**C**) Structure of CFHR3 in complex with the ligand C3b were generated with the DS 3.0. CFHR3 is denoted by pink; C3b (based on the C3 crystal structure, PDB 2WII) consists of a β-chain (residues 1–642) shown in turquoise and α’-chain (residues 730–1641) in steel blue. The binding regions of the CFHR3/C3b complex are presented in yellow. The Arg residue 142 of CFHR3 is highlighted with a CPK model and shown to be directly involved in the binding to C3b.

In detail, the c.424 C > T in the gene CFH3 generates a nonsynonymous mutation p.Arg142Cys, leading to a polar change from a positively charged residue to a neutral one and a moderate decrease of protein isoelectric point (pI) from 7.72 to 7.35. Amino acid (aa) Arg142Cys was located in the CCP2/3 linker. The mutant protein demonstrated a random coil, which was introduced into the amino acid chain in substitution of an original beta-sheet (Fig. 3A). DS 3.0 analysis predicted that the calculated potential energy of the wild type protein is −10569.06 kcal/mol compared to −10542.30 kcal/mol for the mutant one. As substituted by Cys142, the native intramolecular hydrogen bonding interactions between the side chain of Arg142 and neighboring residues (Gly119 and Thr145) were absent (Fig. 3B). These analyses in silico implied that p.Arg142Cys could lead to a decrease in the CFHR3 protein stability.

CFHR3 regulates the complement cascade by binding and interacting with C3b. We then simulated the probable native CFHR3/C3b complex structure. This led to the detection of Arg142 of CFHR3 at the binding interface to C3b (Fig. 3C), indicating its direct participation in the interaction of these two proteins. Our binding free-energy calculation shows a loss of important interaction that p.Arg142Cys declined the binding affinity of CFHR3/C3b complex by 1.26 kcal/mol. Additionally, Align GVGD, SIFT, PROVEAN, SNAP, and PolyPhen-2 independently indicated the deleterious pathogenicity of CFHR3 p.Arg142Cys (Supplemental Table S6).

### Decreased binding to C3b and fluid-phase cofactor activity

**Figure 4:**
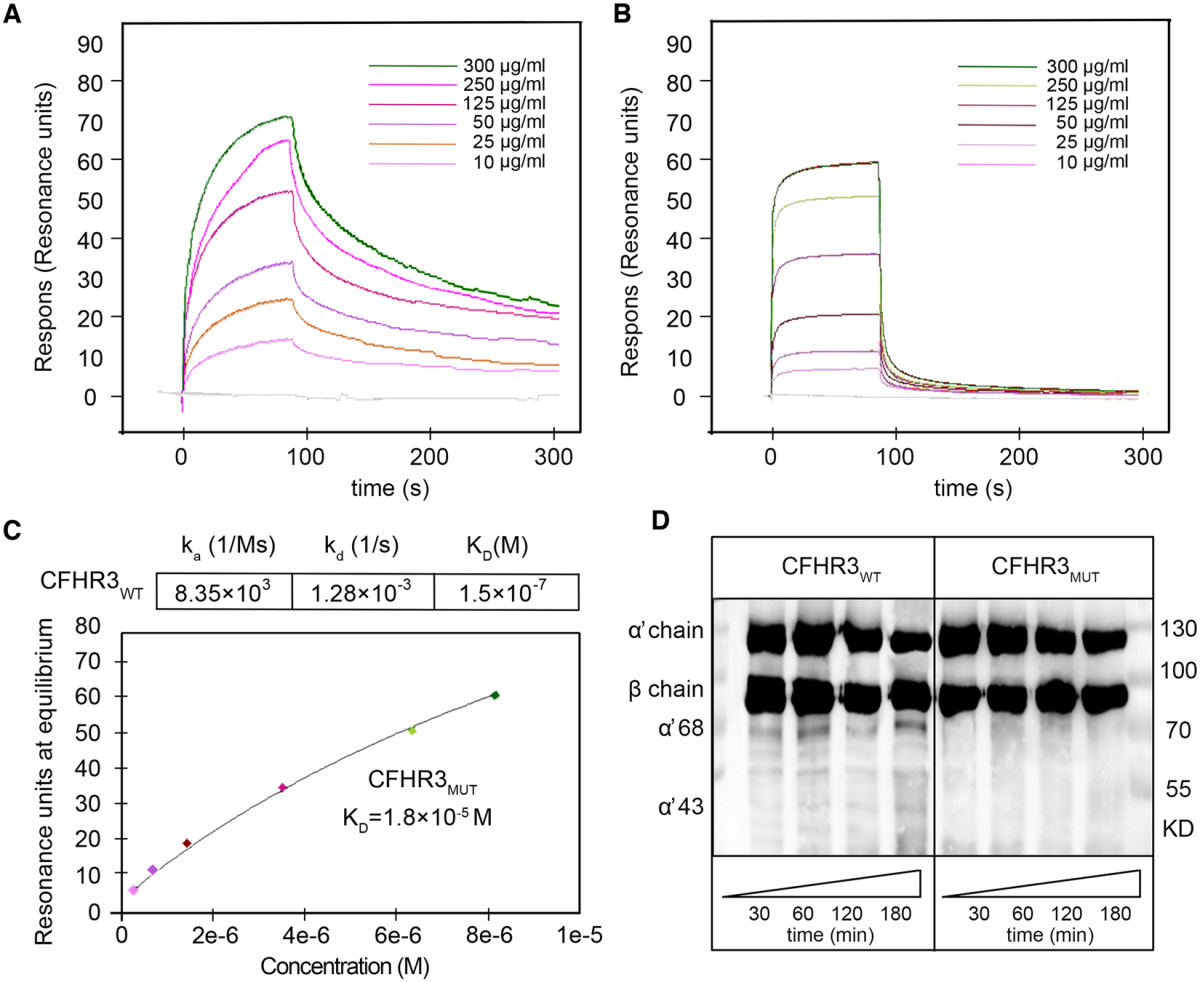
The CFHR3 T risk allele of rs61737525 results decreased binding affinity to C3b and a weaker fluid-phase cofactor activity. C3b was immobilized to a CM5 chip using standard amine coupling. Duplicate injections of CFHR3_WT_ and CFHR3_Arg142Cys_ were performed (concentrations of 10–300 ug/ml) in 10 mM Hepes-buffered saline with 3 mM ENTA and 0.05% (vol/vol) surfactant p20. Overlaid sensograms of binding of (**A**) CFHR3_WT_ and (**B**) CFHR3_Arg142Cys_ to C3b, showing a kinetic and steady-state response, respectively. (**C**) The CFHR3_Arg142Cys_ exhibits a binding to C3b (K_D_ = 1.8 × 10^−5^M; R_max_ = 194.4RU; chi-squared = 0.47), which was lower than that of CFHR3_WT_ (K_D_ = 1.5 × 10^−7^M; R_max_ = 37.2RU; chi-squared = 8.81). (**D**) Limiting concentrations (700 ug/ml) of CFHR3_WT_ and CFHR3_Arg142Cys_ were incubated with factor I and the substrate C3b with increasing time points. Subsequently, the loss of the intact C3b α’-chain and appearance of its factor I cleavage products (43-kDa and 68-kDa fragments) were run on Western blot and visualized. It is apparent that mutant (Arg142Cys) CFHR3 blocks production of cleavage fragments compared to the wild-type one.

To verify the function of Arg142Cys in CFHR3, we generated wild-type (CFHR3_WT_) and mutant (CFHR3_Arg142Cys_) recombinant proteins. Surface plasmon resonance (SPR) showed kinetic response of the wild-type recombinant protein binding to C3b (Fig. 4A) with an association rate constant (k_a_) of 8.35 × 10^3^ (1/Ms) and dissociation rate constant (k_d_) of 1.28 × 10^−3^ (1/s). The equilibrium dissociation constant was calculated from the rate constants as a K_D_ of 1.5 × 10^−7^ M for the CFHR3_WT_ (Fig. 4C). Steady-state response of CFHR3_Arg142Cy_ (Fig. 4B) for C3b was fitted to steady-state affinity measurement, and the K_D_ of CFHR3_Arg142Cys_ − C3b was 1.8 × 10^−5^ M (Fig. 4C). The arg142Cys substitution induced a decrease of approximately two orders of magnitude in the C3b-binding affinity.

Fluid-phase cofactor activity assays of CFHR3_WT_and CFHR3_Arg142Cys_ were further undertaken to show the functional activity as a cofactor for factor I-mediated cleavage of C3b (Fig. 4D). With CFHR3_WT_, C3b showed two proteolytic fragments of 68 kDa and 43 kDa, referred to as α’-chain of C3b degradation, which became obvious as incubation time increased. However, cleavage fragments were not clearly visible when CFHR3-Arg_142_ acted as a cofactor. These findings suggested that the CFHR3_Arg142cys_ variant was less efficient as a cofactor for factor I in the cleavage of C3b in the fluid phase relative to the wild-type one.

## Discussion

The alternative pathway (AP) of the complement system is a powerful and evolutionarily old defense system of innate immunity that recognizes and destroys invading infectious microbes and also targets and eliminates modified self-cells. The AP is a spontaneous self-amplifying initiator of complement, and is activated by default when blocked by inhibitors^17–19^. Defective AP regulation often results in severe autoimmune diseases. Recent evidence shows that mutations and sequence variations of important regulators of AP cause a variety of kidney diseases in the form of aHUS and C3G, including DDD, C3GN, and MPGN I^11, 20–22^.

In the present study, we simultaneously identified three rare SNPs, rs55807605 (CFH c.2509 G > A), rs61737525 (CFHR3 c.424 C > T), and rs57960694 (CFHR5 c.434 G > A), in four sporadic cases with different phenotypes of these diseases. They were all nonsynonymous mutations and rs55807605 was previously reported in a Japanese patient with aHUS, without definite clinical significance and functional studies^23^. All these SNPs were located in the genes of members of the complement factor H family where FHRs act around or in concert with the central AP regulator complement factor H (CFH).

The human factor H protein family is encoded by six genes positioned in tandem on the human chromosome 1q31.3 within the regulator of complement activation (RCA) gene cluster. This family includes CFH, as well as five CFH-related proteins: CFHR1, CFHR2, CFHR3, CFHR4 and CFHR5. A general role for the factor H protein family in the regulation of complement activation is emerging, and genetic deficiency of this family has been associated with aHUS, C3G, and MPGN I^24–27^. FH-HUS web database (http://www.FH-HUS.org) provides 193 mutations (mainly including missense, nonsense, and polymorphisms) in CFH; mutations in FHR proteins are also recorded elsewhere, but in low occurrences.

CFH family genes are supposed to be highly linked. With available databases, 15 common SNPs separated by 272.6 kb in the CFH gene cluster were adequately covered by strong linkage disequilibrium (LD). The three SNPs, rs55807605, rs61737525 and rs5796069, are also in perfect LD according to our research group observations and those of public cohorts. Two haplotypes were identified in the CFH gene cluster region (GCG and ATA). Besides, multiple independent large genomic duplications within the gene cluster, also known as low-copy repeats, make it highly prone to genomic rearrangements through gene conversion and non-allelic homologous recombination. The frequently observed genomic rearrangements of CFH/CFHR locus were actually identified in individuals with these rare renal diseases. However, potential genetic fusion linked with the three SNPs have been excluded by qPCR and western blot assays in the present study.

The four patients with rs55807605, rs61737525 and rs57960694 all carried ATA haplotype, with an estimated population frequency of 1.2% in the Chinese Han population (1000 Genomes Project Phase 3), as well as three common haplotypes (CTTCT, CAGGAAT and TTA). It indicates a close genetic link of mutation and alleles of CFH/CFHR genes, meaning they are inherited together. Haplotype-association analysis further revealed that ATA (A at rs55807605, T at rs61737525, and A at rs57960694) is significantly associated with increased risk (P = 0.029; OR, 6.72; 95% CI, 1.22–36.99). Moreover, the allele T of SNP rs61737525 can be as a marker for the risk haplotype, with an OR of 4.21 (CI = 1.12–15.84 P = 0.036). It demonstrated that rs61737525 (CFHR3 c.424 C > T) might be a leading pathogenic variant in this risk locus.

Because the majority of risk variations in complement genes lack experimental verification, the potential pathogenic effect is difficult to be confirmed. Existing verified functional mutations or polymorphisms cluster in the C-terminal region (Glu1172Stop, Arg1210Cys, Arg1215Gly)^15, 28^, or in the N-terminal region (Arg53His, Ile62Val, Arg78Gly, Arg83Ser)^29–31^ of the complement regulator factor H. In our research, the potential pathogenicity of the causative missense mutation c.424 C > T (p.Arg142Cys) in the gene CFHR3 is predicted to be deleterious by multiple prediction tools. The change in nature and size of residue alters local structural properties from an original beta-sheet to a random coil. This may be due to the loss of intramolecular hydrogen bonds between the side chain of Arg142 and its neighboring residues. Accordingly, the mutant protein CFHR3_Arg142Cys_ is predicted to be destabilized as compared to the wild-type protein CFHR3_WT_ based on energy parameters.

CFHRs are immunologically and structurally related to factor H but their functions have not yet been well established at present. Surface plasmon resonance techniques have suggested recombinant CFHR3 bind to C3b, exhibiting low cofactor activity for factor I in the cleavage of C3b^32^. Based on homology modeling, Arg142 was predicted to be located at the binding interface to C3b to participate in the protein-protein interaction. The Arg142Cys substitution destabilized the protein-protein interaction of the CFHR3/C3b complex by 1.26 kcal/mol. Encouragingly, the above algorithms used to predict the molecular pathomechanism were found to be in a good agreement with our experimental data: the affinity of CFHR3_Arg142Cys_ for C3b was estimated to be weak compared with CFHR3_WT_ by the SPR analysis, in accordance with the declined cofactor activity in the fluid phase. In addition, binding of the factor H family proteins to polyanions such as heparin is presumed to contribute substantially to the discrimination between activator and non-activator surfaces, but binding energy calculation in silico prediction prompted that this substitution is insufficient to affect the binding of CFHR3 to heparin (Supplemental Table S7).

Notably, a pathogenic haplotype in the CFH gene family is associated with different kidney diseases. Several interpretations could explain this issue. First, additional genetic factors or mutation patterns might contribute to the distinct phenotype, as the complement system is integrated with numerous signaling pathways. Second, the presence of acquired AP abnormalities, including C3Nefs, anti-CFH autoantibodies, and monoclonal proteins, may be responsible for the disease progression. Finally, additional triggers (e.g., environment, drugs, pregnancy, infection, etc.) could be critical predisposing factors and influence the pathologic outcome in the context of case histories with similar genetic background.

One point should be noted that the relatively small size in the present study may reduce the statistical power. Rs55807605, rs61737525 and rs57960694 are all located in contiguous regions with similar origins and functions, which may directly contribute to the impaired complement function, and rs61737525, a cysteine at amino acid 142, leads to decreased binding to C3b and cofactor activity compared with wild-type CFHR3 protein in which arginine is at this position. However, we could not rule out whether the other two SNPs add to such dysfunction or they are genetically linked with rs61737525 without biological function. It is also problematic whether this haplotype is genetically linked with another risk-related one and only slightly enhances disease susceptibility.

To summarize, our study firstly reports the case of a shared genetic background within the CFH gene cluster among aHUS, DDD, C3GN and MPGNI. The leading pathogenic variation in this risk haplotype ATA is CFHR3 p.Arg142Cys (rs61737525), which impairs its C3b-binding properties and cofactor activity in the fluid phase. Expanding our knowledge on the genetics, tissue expression and physiological functions of the CFHR proteins will be of great value to the understanding of these severe anomalies, and hopefully—to more beneficial treatment of AP abnormalities.

## Material and Methods

### Subjects

A total of 91 patients (mean age 40.4 ± 15.9 years, range 13–80 years) with rare nephropathies enrolled from the Renal Biobank of the National Clinical Research Center of Kidney Diseases, Jinling Hospital, Nanjing University School of Medicine, PR China. They were all Han Chinese from the eastern area of China, including 10 patients with DDD, 33 with C3GN, 24 with MPGN I and 24 with aHUS. The diagnosis of G3GN, DDD, MPGNI and aHUS was established based on clinical characteristics and renal lesions per clinical records. Serologic tests for hepatitis B or C and cryoglobulinemia were negative in all patients, and there was no history of autoimmune disease, long-term infection, or monoclonal gammopathy. Meanwhile, 300 healthy age-matched controls were recruited from a panel of unaffected, genetically unrelated Han Chinese individuals from the same geographic region. The demographic characteristics of patients and controls are presented in Supplemental Table S1.

Written informed consent forms were obtained from all participants. All experiments protocols were approved by the Ethics Committee of the Jinling Hospital (Nanjing, China) in accordance with the Declaration of Helsinki. All methods were conducted according to the manufacturers’ instructions and in strict accordance with the recommendations in the guidelines set forth by the Ethics Committee of the Jinling Hospital (Nanjing, China).

### Targeted exon sequencing and data analysis

Genomic DNA was isolated from 2 ml peripheral blood of each subject using the Wizard Genomic DNA Purification Kit (Promega, USA) following the manufacturer’s instructions. RefSeq coding exons from 11 reported disease-related complement genes were targeted for sequencing, including C3 (NM 000064), MCP (NM 002389), CFB (NM 001710), CFH (NM 000186), CFHR1 (NM 002113), CFHR2 (NM 005666), CFHR3 (NM 021023), CFHR4 (NM 006684), CFHR5 (NM 030787), CFI (NM 000204) and THBD (NM 000361). The approximately 92.96% of reads were mapped to the target regions at an average of 508×, and sequencing results were credible (Supplemental Table S2). Detailed information for capture design, sequence capture, library preparation and Ion Torrent sequencing has been previously described^33, 34^.

### Genotyping

Pyrosequencing was to assess genotyping of rs55807605, rs61737525 and rs57960694. All primers including both the PCR and sequencing primers were designed using PyroMark Assay Design (2.0; Qiagen, Venlo, the Netherlands) and shown in Supplementary Table S3. The DNA amplification, product processing and run were operated using the PyroMark Q96 ID System (Qiagen).

The public database 1000 Genomes Project Phase 3 provided screening data of rs55807605, rs61737525 and rs57960694 and other 15 common SNPs, namely rs1061147 (SNP 1), rs1061170 (SNP 2), rs203674 (SNP 3), rs390154 (SNP 4), rs400642 (SNP 5), rs409549 (SNP 6), rs409253 (SNP 7), rs424925 (SNP 8), rs42472 (SNP 9), rs399507 (SNP 10), rs438781 (SNP 11), rs4230 (SNP 12), rs3828032 (SNP 13), rs3748556 (SNP 14), and rs3748555 (SNP 15) were further collected based on the 1000 Genomes Project (May 2013) and analyzed as a general population control.

### Haplotype analysis

Construction of block structures with distribution of haplotypes was accomplished using Haploview 4.2 (www.broadinstitute.org/haploview/haploview). With the Haploview program, linkage disequilibrium (LD) plots and the corresponding population frequencies were shown.

### Real-time quantitative PCR

Real-time quantitative PCR was undertaken to determine copy number, relative to the nuclear GAPDH gene, using three pairs of primers specific to an RS gene locus (rs55807605, rs61737525, rs57960694). Sequences for the primers whose positions are shown in Fig. 2A are given in Supplemental Table S4.

### Western blot analysis

Plasma samples were subjected to the SDS-PAGE using 6% and 10% gels. Western blotting was performed using polyclonal rabbit anti human complement factor H antiserum (Sigma-Aldrich, USA) and polyclonal rabbit anti human CFHR3 antiserum (Proteintech, USA).

### Homology modeling and molecular docking

The X-ray or NMR structure of CFHR3 protein has not yet been successfully determined. Starting from the amino acid sequence 19–330 (AAH58009.1), 3D homology-modeled structures of wild-type and mutant CFHR3 were first generated by an integrated platform for automated protein structures called iterative threading assembly refinement (I-TASSER) server (http://zhanglab.ccmb.med.umich.edu/I-TASSER/)^35^. The one with the highest value of C-score was selected to be refined closer to the native structure by a molecular dynamics (MD) tool called Fragment-Guided MD simulation (FG-MD)^36^. Potential energy of refined protein was estimated by “Calculate Energy” protocol of Discovery Studio (DS) 3.0. C3b (template) (PDB ID: 2WII) structure was obtained from protein data bank (www.rcsb.org/pdb).

Energy minimization of the protein structure was performed by applying “Prepare Protein” protocol of DS. This protocol cleans the protein molecule by adding missing atoms, inserting missing loops, assigning charges and fixing CHARMm force fields. A total of 2000 docking poses were generated by ZDOCK (CHARMm-based DOCKER) protocol and incorporated within DS 3.0. The No.1 ranked model was considered as the probable complex structure. Binding free energy simulation of protein-protein interaction was estimated by the Calculate Mutation Energy (Binding) protocol. Binding energy of protein-small molecule (heparin, PDB ID: 1HPN) was calculated by the Calculate Binding Energies protocol. Visualization of hydrogen bonding networks was demonstrated by Molecular Operating Environment (MOE) (http://www.chemcomp.com/MOE-Molecular_Operating_Environment.htm).

### Evaluation of pathogenicity

The ProtParam tool (http://web.expasy.org/protparam/) was used to compute various physical and chemical parameters including the molecular weight and theoretical pI. The Align GVGD (http://agvgd.iarc.fr/), SIFT (http://sift.jcvi.org/), PROVEAN (http://provean.jcvi.org/index.php), SNAP (http://rostlab.org/services/snap/), and PolyPhen-2 (http://genetics.bwh.harvard.edu/pph2/dbsearch.shtml), were utilized to predict the effect of mutation impact on protein function. Possible gene-gene interactions were determined using Multifactor Dimensionality Reduction (MDR) (http://www.epistasis.org).

### Recombinant proteins and purified proteins

The genes encoding the wild-type and mutant CFHR3 were performed by codon optimization and synthetized. The synthetic genes were subcloned into the vector pET-28a (+) and the plasmids were transformed into *E. coli* strain BL21 (DE3) as previously described^37^. Chromatography was performed on the HPLC™ System using a Ni2+ -charged, 1 ml HiTrap Chelating HP column. Total protein staining with Pierce Coomassie Brilliant Blue 250 (Thermo-Scientific, USA) of the eluted fraction was used for purity determination. C3b and factor I, purified from normal human serum, were all purchased from CompTech (Complement Technology, Inc., Tyler, TX, USA).

### Surface plasmon resonance-binding assays

The resonance binding of recombinant CFHR3_WT_ and CFHR3_Arg142Cys_ was assayed using a Biacore T200 instrument (GE Healthcare)^31, 38^. Approximately 2488 resonance units of C3b (CompTech) were immobilized via a standard amine coupling procedure on a Biacore series CM5 sensor chip (GE Healthcare). Experiments were performed at 25 °C and a flow rate of 30-ul/min. Data were processed using the Biacore T200 Evaluation Software (GE Healthcare). The affinity of an interaction (equilibrium dissociation constants, K_D_) was determined from the dependence of steady-state binding levels on analytic concentrations, or calculated as the ratio between kinetic rate constants (https://www.biacore.com/lifesciences/help/basic_theory_of_affinity/).

### Cofactor activity assay in fluid phase

Kinetic fluid-phase assays were used to measure the cofactor activity for proteolytic cleavage of C3b by factor I. C3b, factor I and CFHR3_WT_/CFHR3_Arg142Cys_ were incubated at 37 °C for 30, 60, 120, and 180 minutes. The reaction was stopped with the addition of 5× sample loading buffer and then electrophoresed on 8% gels. After transfer to a nitrocellulose membrane, proteins were visualized with goat anti-C3 antibody (1:64000; CompTech, Inc.).

### Statistical analysis

All tests were performed using SPSS statistical software (version 20; SPSS Inc., Chicago, IL). The Pearson chi-square (χ^2^) or Fisher’s exact test was used to assess allelic associations, genotypic association and haplotype association statistics. OR and 95% CIs were also calculated. A P value < 0.05 was considered as statistically significant.

## Electronic supplementary material

Supplementary Info
