# Supplementary material for: A haplotype in CFH family genes confers high risk of rare glomerular nephropathies
Source: Sci Rep. 2017 Jul 20;7:6004. doi: 10.1038/s41598-017-05173-8 (PMC5519609; doi:10.1038/s41598-017-05173-8)

Supplementary information for:

A haplotype in CFH family genes confers high risk of rare glomerular nephropathies

Yin Ding[1,2], Weiwei Zhao[2], Tao Zhang[2], Hao Qiang[3], Jianping Lu[1,2], Xin Su[3], Shuzhen Wen[2], Feng Xu[2], Mingchao Zhang[2], Haitao Zhang[2], Caihong Zeng[2], Zhihong Liu[1,2*], and Huimei Chen[2*]

[1]Devision of Nephrology, Jinling Hospital, Southern Medical University, Nanjing, China, 210016

[2]National Clinical Research Center of Kidney Diseases, Jinling Hospital, Nanjing University School of Medicine, Nanjing, China, 210016

[3]Center of Drug Discovery, State Key Laboratory of Natural Medicines, China Pharmaceutical University, Nanjing, China, 210009

[*]To whom correspondence and request for materials should be addressed.

Supplementary Tables (on following pages)

- **Supplementary Table S1.** Demographic distribution of the Study Population
- **Supplementary Table S2.** Quality of targeted resequencing results
- **Supplementary Table S3.** Primers for pyrosequencing of point mutations
- **Supplementary Table S4.** Primers of qPCR for copy number
- **Supplementary Table S5.** Clinical features and laboratory evaluation

• **Supplementary Table S6.** C.424 C>T variant in CFHR3 analyzed by five publicly available programs (SIFT, SNAP, Align GVGD, PROVEAN and PolyPhen-2)

• **Supplementary Table S7.** Comparison of binding energy of wild-type and mutant CFHR3 to heparin

• **Supplementary Figure S1.** Interaction dendrogram for rs55807605, rs61737525 and rs57960694 by the MDR

Supplemental Table S1. Demographic distribution of the Study Population

**Study population（n）**	**Age(mean ± SD)**	**Age Range**	**Gender(female/male)**
Controls(300)	39.5±10.9	20-64	201(67.0%)/99
Total cases(91)	40.4±15.9	13-80	35(38.5%)/56

n = number of subjects; SD = standard deviation.

Supplemental Table S2. Quality of targeted resequencing results

	**Total 91patients (DDD =10, C3GN=33, MPGNI n=24, aHUS n=24)**
Total number of Bases (Mbp)	4386.56
▸ Number of Q20 Bases(Mbp)	3697.63
Total number of Reads	32,342,947
Mean length (bp)	134
Longest Read (bp)	372
TF (Test Fragments) Percent	87%~94%

Q20=bases of Q>=20 / all bases of sequencing

Supplemental Table S3. Primers for pyrosequencing of point mutations

	**Forward primer**	**Reverse primer**	**Sequencing primer**
CFH c.2509G>A	ATTATGCCCACCTCCACC	GGTATTGACTGCCATCTTCC	GGGATGGAGAAAAAGT
CFHR3 c.424C>T	GAAGTTGCCTGCCATCCT	TGAGACTGTCGTCCGTGTTA	TCCTACTCCCAGATGC
CFHR5 c.434G>A	TACTAGCGGTTTTACGGGCG	TCGAACAGGAGGAGCAGAGAGCGA	AAATTGGAACATGACAT

Supplemental Table S4. Primers of qPCR for copy number

	Forward primer	Reverse primer	PCR product size (bp)
CFH	ATTATGCCCACCTCCACC	GGTATTGACTGCCATCTTCC	159
CFHR3	GAAGTTGCCTGCCATCCT	TGAGACTGTCGTCCGTGTTA	166
CFHR5	ACTCTGTATATGAAGCCCCT	TTTCAACACGTCTCCAACTT	187
GAPDH	TACTAGCGGTTTTACGGGCG	TCGAACAGGAGGAGCAGAGAGCGA	166

Supplemental Table S5. Clinical features and laboratory evaluation

**Patient**	**Age/sex at presentation**	**Serum creatinine at diagnosis (mg/dl)**	**Urinary protein (g/d)**	**C3/C4 (g/l)**	**Renal outcome**
1	28/F	9.44	Anuria	0.58*/0.28	ESRD
2	23/M	0.79	5.99	0.17*/0.18	CKD1
3	12/F	0.62	9.56	1.08/0.32	ESRD
4	34/F	0.58	1.19	1.00/0.24	CR

Abbreviations: C3, normal range 0.8-1.8 g/l; C4, normal range 0.1-0.4g/l. * Low.
ESRD, eGFR<15ml/min/1.73m^2 or requirement of dialysis; CR, complete remission.

Supplemental Table S6. C.424 C>T variant in CFHR3 analyzed by five publicly available programs (SIFT, SNAP, Align GVGD, PROVEAN and PolyPhen-2)

**Gene**	**Variant**	**SIFT**	**SNAP**	**Align GVGD**	**PROVEAN**	**PolyPhen-2**
CFHR3	c.424C>T p.R142C	Damaging	Effect	Most likely to interfere with function	Deleterious	Probably damaging

Supplemental Table S7. Comparison of binding energy of wild-type and mutant CFHR3 to heparin

**Receptor / Ligand**	**Binding Energy (kcal/mol)**	**Complex Energy (kcal/mol)**	**Entropic Energy (kcal/mol)**
$CFHR3_{WT}$/Heparin	-181.52265	-9472.91760	26.67120
$CFHR3_{Arg142Cys}$/Heparin	-181.52252	-9446.15087	26.64910

Supplemental Figure S1. Interaction dendrogram for rs55807605, rs61737525 and rs57960694 by the MDR. Red or orange lines indicate strong synergistic interaction; blue or green lines indicate redundant interaction or no interaction.

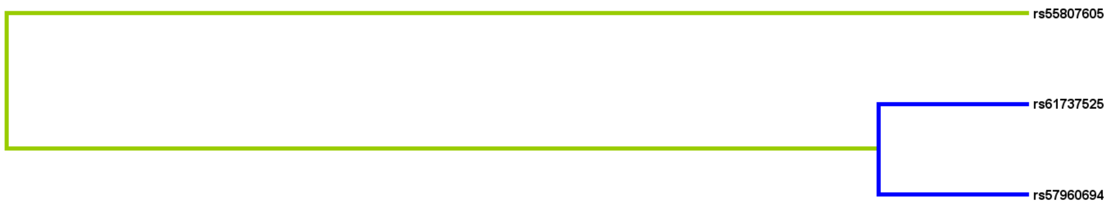

Supplement: Supplementary file 1 — Supplementary Info [file 41598_2017_5173_MOESM1_ESM.pdf]
